# Design of Temperature-Responsive Cell Culture Surfaces for Cell Sheet Engineering

**DOI:** 10.34133/2021/5738457

**Published:** 2021-02-03

**Authors:** Y. Akiyama

**Affiliations:** Institute of Advanced Biomedical Engineering and Science, Tokyo Women's Medical University, TWIns, Tokyo, Japan

## Abstract

Temperature-responsive cell culture surfaces, which modulate cell attachment/detachment characteristics with temperature, have been used to fabricate cell sheets. Extensive study on fabrication of cell sheet with the temperature-responsive cell culture surface, manipulation, and transplantation of the cell sheet has established the interdisciplinary field of cell sheet engineering, in which engineering, biological, and medical fields closely collaborate. Such collaboration has pioneered cell sheet engineering, making it a promising and attractive technology in tissue engineering and regenerative medicine. This review introduces concepts of cell sheet engineering, followed by designs for the fabrication of various types of temperature-responsive cell culture surfaces and technologies for cell sheet manipulation. The development of various methods for the fabrication of temperature-responsive cell culture surfaces was also summarized. The availability of cell sheet engineering for the treatment and regeneration of damaged human tissue has also been described, providing examples of the clinical application of cell sheet transplantation in humans.

## 1. Introduction

Cell-based medicine has been expected as a promising approach to treat damaged tissues and organs. Langer and Vacanti proposed tissue engineering as a new research field by synergistically combining engineering and life sciences [1]. Their concept initiated the development of functional substitutes for damaged tissues and organs. Tissue engineering involves cell culturing on synthetic and/or natural polymeric porous scaffolds in the presence of growth factors. Some of the engineered tissues were successfully applied to the treatment of damaged tissue and/or tissue fabrication [2–5]. The engineered tissues and organs were constructed by seeding and culturing cells on a biodegradable scaffold. The immigrated and/or proliferated cells and their secreted extracellular matrix (ECM) filled spaces where the scaffold was degraded during the cell culture. Through these complicated processes, cells assemble and form three-dimensional (3D) tissue-like structures themselves. Adjustment of speed between scaffold degradation and space filling with cells was required for subsequent assembly and formation of the engineered 3D tissue. However, the adjustment remained difficult. In addition, in the deep part of the porous scaffold, diffusion of O_2_ and nutrients, required for the survival of cells, was limited, possibly preventing cell migration toward the deep part. Therefore, the resulting engineered cultures have a lower cell density, different from native tissue with high cell density, such as the muscles and heart. In addition, acidic components degraded from the synthetic scaffold caused a nonspecific inflammation response after implantation, damaging the cells [6].

In contrast with conventional tissue engineering, Okano's group has shown a novel approach: cell sheet engineering, which deals with fabricating, manipulating, and transplanting cell sheets [7, 8]. A cell sheet was fabricated with an intelligent nano-bio-interface, temperature-responsive cell culture surface [9, 10]. Cell sheet engineering enables the assembly of 3D and cell-dense tissue-like structures without scaffolds by layering the cell sheet. The cell sheet and/or layered cell sheets facilitate transplantation to biological tissues and organs without the need for sutures. Such transplantable cell sheets have been clinically applicable for the regeneration and treatment of damaged human tissues and organs such as the heart [11], corneal epithelium [12], esophagus epithelium [13, 14], periodontal ligaments [15, 16], cavity of the middle ear [17], knee cartilage [18], and lungs [19]. Cell sheet engineering is potentially promising in both tissue engineering and regenerative medicine.

## 2. Temperature-Responsive Cell Culture Surface and Cell Sheet

### 2.1. Features of Temperature-Responsive Cell Culture Surfaces and Cell Sheet Recovered from the Surfaces

**Figure 1:**
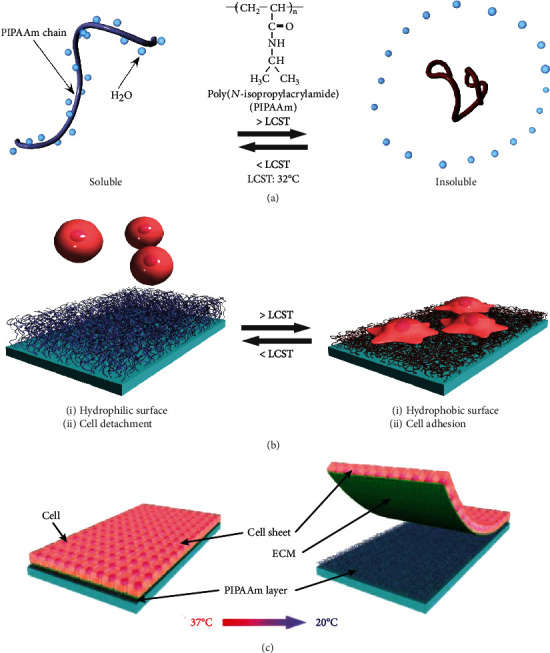
Illustration of characteristics of (a) poly(*N*-isopropylacrylamide) (PIPAAm), (b) temperature-responsive cell culture surface, PIPAAm-TCPS, and (c) cell sheet recovery from PIPAAm-TCPS. (a) PIPAAm chains show reversible temperature-dependent hydration and dehydration across LCST. (b) Above LCST, the grafted PIPAAm chains dehydrated to exhibit enough hydrophobic and cell adhesion properties (right). Below LCST, cells were detached spontaneously from the surface due to hydration of grafted PIPAAm chains (left). These images of (a) and (b) from reference [36] (Z. Tang and T. Okano., Recent development of temperature-responsive surfaces and their application for cell sheet engineering, Regen Biomater, 1, pp. 91-102) are under a Creative Commons Attribution 4.0 International License (CC-BY-4.0). (c) After adhered cells were confluent at 37°C (left), lowering the temperature to 20°C (below LCST) enabled the detachment of the cell sheet from PIPAAm-TCPS, preserving the deposited ECM and cell-cell junctions (right). Reprinted permission from reference [8]. Copyright © 2021, Oxford University Press.

A temperature-responsive polymer, poly (*N*-isopropylacrylamide) (PIPAAm), was synthesized in the latter half of the 1960s. The phase transition of PIPAAm in an aqueous solution was reported for the first time (Figure 1(a)) [20]. When temperatures decrease across 32°C (lower critical solution temperature (LCST) of PIPAAm), PIPAAm is dissolved and expands its chain conformation due to hydration of PIPAAm chains. In contrast, PIPAAm chains are quickly aggregated and insoluble because of the dehydration of PIPAAm chains when the temperature reaches above the LCST. The temperature-dependent hydration and dehydration are reversible.

Approximately 30 years later, by grafting PIPAAm on a tissue culture polystyrene surface (PIPAAm-TCPS), Okano et al. successfully invented temperature-responsive surfaces for the first time, in which hydrophilic and hydrophobic properties are modulated with temperature (Figure 1(b)) [9]. They grafted PIPAAm gel on a tissue culture polystyrene (TCPS) surface with electron beam (EB) irradiation and exploited PIPAAm-TCPS as a cell culture surface [9]. They showed that bovine hepatocytes adhere to PIPAAm-TCPS at 37°C, where PIPAAm-TCPS surface became hydrophobic due to dehydration of PIPAAm chains. The adhered cells spontaneously detached at 20°C, where PIPAAm-TCPS are hydrophilic because of hydration of grafted PIPAAm chains (Figure 1(b)). PIPAAm-TCPS enables the modulation of cell adhesion/detachment characteristics through mild temperature changes.

Okano's group intensively investigated cell adhesion and detachment mechanisms in terms of the cellular metabolic system [21, 22]. They found that cell adhesion to the PIPAAm-TCPS surface was involved in cellular metabolic processes as well as cytoskeletal reorganization, referred to as active adhesion. Adhered cells proliferate to confluency on PIPAAm-TCPS surface. During cell culture, adhered cells secrete ECM such as fibronectin [23]. After confluency, the cells were detached from PIPAAm-TCPS surface as a monolayer sheet by lowering the temperature below LCST (Figure 1(c)). Conventionally, trypsin and/or EDTA treatment is commonly employed for recovering adhered cells from cell culture surfaces, destroying cell-ECM interactions and cell-cell junctions. In contrast, the low-temperature treatment avoids destruction, such as the cell-ECM interaction and cell-cell junction, enabling recovery of cell sheet, thereby preserving the secreted ECM and cell-cell junctions (Figure 1(c)). As the deposited ECM functions as a biological glue, recovered cell sheets are readily layered onto other cell sheets for the fabrication of 3D tissues that are subsequently transplanted [24, 25].

### 2.2. Design of Grafted PIPAAm Layer for Recovering Cell Sheet as a Temperature-Responsive Cell Culture Surface

**Figure 2:**
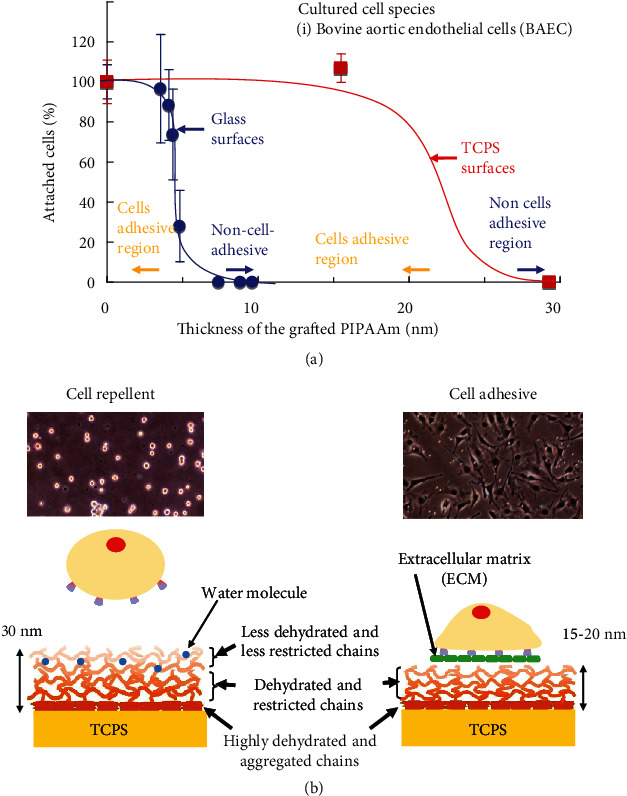
Graft polymer thickness dependency on the resulting cell adhesion character. (a) Correlation between the percentage of adhered cell and thickness of the grafted PIPAAm gel layer. Blue circles and red squares represent PIPAAm-GC and PIPAAm-TCPS with various PIPAAm graft gel thickness, respectively. Reprinted permission from reference [28]. Copyright © 2010,WILEY-VCH Verlag GmbH & Co. KGaA, Weinheim. (b) Illustration of graft polymer thickness dependency of PIPAAm-TCPS. PIPAAm-TCPS with 2.9 *μ*g/cm^2^ (left) and 1.4 *μ*g/cm^2^ (right) of PIPAAm graft density (right). At the interface of basal hydrophobic TCPS surface, grafted PIPAAm chains are dynamically restricted, highly aggregated, and dehydrated due to the TCPS surface. Such aggregation and dehydration successively dehydrated PIPAAm chains at the outermost surfaces, which allow the surfaces to be cell adhesive at 37°C (right). In the case of the surfaces thicker than 20 nm, the aggregation and dehydration do not effectively dehydrate PIPAAm layer in the outermost surfaces region (left). The outermost surface is more hydrated than thinner PIPAAm-TCPS. Resulting temperature-responsive cell culture surface shows a cell repellent character. Reprinted with permission from reference [27]. Copyright © 2018, The Society of Polymer Science, Japan.

It was observed that the grafted PIPAAm layer of PIPAAm-TCPS likely affected subsequent cell adhesion behavior, whereas the surface of the macroscopic PIPAAm gel, prepared using IPAAm and a cross-linking agent, did not exhibit cell adhesive character [10, 26]. To investigate the effects of the grafted PIPAAm layer on the resultant cell adhesive character, two types of PIPAAm-TCPS with different thicknesses of the graft PIPAAm gel were prepared and characterized [10, 27]. The thicknesses of the grafted PIPAAm layers were directly measured by use of a UV excimer laser and an atomic force microscope (AFM) and were found to be 20 nm (20PIAAm-TCPS) and 30 nm (30PIPAAm-TCPS). Bovine carotid artery endothelial cells did not attach to 30PIPAAm-TCPS at 37°C, where the PIPAAm chains are dehydrated. By contrast, cells adhered to the 20PIPAAm-TCPS as well as TCPS at 37°C. At a lower temperature of 20°C, the adhered cells were completely detached from 20PIPAAm-TCPS. The fact that adsorbed fibronectin was not detected on 30PIPAAm-TCPS supported that 30PIPAAm-TCPS was a non-cell-adhesive surface. The grafted polymer thickness affects the subsequent cell attachment behavior. The graft polymer-thickness dependency on cell adhesion property was also observed in PIPAAm-grafted glass surface (PIPAAm-GS). The correlation between the graft polymer thickness and cell adhesion ratio was graphically plotted (Figure 2(a)) [28]. In the case of PIPAAm-GS, a 3.3 nm-thick grafted PIPAAm layer (3.3PIPAAm-GS) was optimal for expressing the temperature-dependent cell attachment/detachment. When the thickness was greater than the optimal polymer thickness (e.g., 8.8 nm-thick grafted PIPAAm layer (8.8PIPAAm-GS)), cells were not adhered to PIPAAm-GS even at 37°C, similar to that observed in 30PIPAAm-TCPS [10]. The dependency of graft polymer thickness on cell adhesion is explained in terms of the degree of molecular mobility and dehydration of graft PIPAAm chains [10, 28]. This consideration is illustrated in Figure 2(b) [27]. The configuration and molecular mobility of the grafted PIPAAm chains influence the surface wettability with temperature changes [29–31]. This consideration was applied to the nanoscale PIPAAm-grafted layer of PIPAAm-TCPS and PIPAAm-GS surfaces. Hydrophobic and immobile basal TCPS surface restrict grafted PIPAAm chains close to the TCPS surface, promoting aggregation and dehydration of grafted PIPAAm chains. Such restriction and dehydration of the grafted PIPAAm chains successively promote dehydration at the outermost surface of the 20PIPAAm-TCPS chains, consequently providing 20PIPAAm-TCPS with cell adhesive character. In contrast, in the case of 30PIPAAm-TCPS with the thicker polymeric layer, the successive restriction and dehydration of PIPAAm chains at the basal TCPS did not substantially affect the dehydration of grafted PIPAAm chains at the outermost regions. Polymer chains in the outermost regions of 30PIPAAm-TCPS are more hydrated than those on 20PIPAAm-TCPS. Whereby, cells do not attach to the thicker 30PIPAAm-TCPS at 37°C.

AFM observation of the increase and decrease in swelling of the graft PIPAAm layer of 3.3PIPAAm-GS and 8.8PIPAAm-GS at 25°C and 37°C, respectively, under aqueous conditions strongly supported the characteristics of the nanoscale PIPAAm graft layer as described above [28]. Immersing 3.3PIPAAm-GS in an aqueous solution increase the thickness from 3.3 nm ± 0.3 nm (dry state) to 8.0 nm ± 1.4 nm (25°C) and 6.0 ± 1.4 nm (37°C). The thickness of the graft PIPAAm layer was not substantially altered between above and below the LCST. The graft PIPAAm layer of 3.3PIPAAm-GS possibly maintained an unswollen state under aqueous conditions irrespective of temperature. By contrast, 8.8PIPAAm-GS shows a substantial increase of the graft PIPAAm layer, 25.4 nm ± 4.1 nm (25°C) and 12.9 ± 2.2 nm (37°C) in aqueous conditions, depending on temperature change. The PIPAAm graft layer of 8.8PIPAAm-GS exhibited swelling and deswelling behavior by temperature change. These results indicate that grafted PIPAAm chains in the thinner PIPAAm layers are readily subjected to successive dehydration and molecular restriction from the grafted polymer chains, which are close to the basal surface, as illustrated in Figure 2(b). The design of the nanoscale PIPAAm layer has contributed to the development of various methods for fabrication of temperature-responsive cell culture surfaces using various methods, as described below (“Development of Various Methods for Fabrication of Temperature-Responsive Cell Culture Surfaces”).

## 3. Design of a Temperature-Responsive Cell Culture Surface for Rapid Recovery of Cell Sheets

Cell sheet detachment from the PIPAAm-TCPS proceeds slowly and 20–30 min or more is required for complete recovery of cell sheet. As hydration of grafted PIPAAm chains gradually proceed from the interior fringe of PIPAAm-TCPS toward the center, the cell sheet also gradually detaches from the sheet fringe. Rapid recovery of cell sheet is desirable for reducing the time of subsequent cell sheet transplantation as well as the patient's burden. It also shortens the time required for layering of cell sheets, leading to shortened times for assembling 3D tissue and organs. Therefore, temperature-responsive cell culture surfaces for rapid cell sheet detachment are an attractive tool. To date, several temperature-responsive cell culture surfaces have been designed to promote the hydration of grafted PIPAAm chains followed by rapid cell sheet detachment.

### 3.1. Temperature-Responsive Cell Culture Surface Using Microporous Membrane and/or Incorporating Hydrophilic Polymeric or Monomeric Components

**Figure 3:**
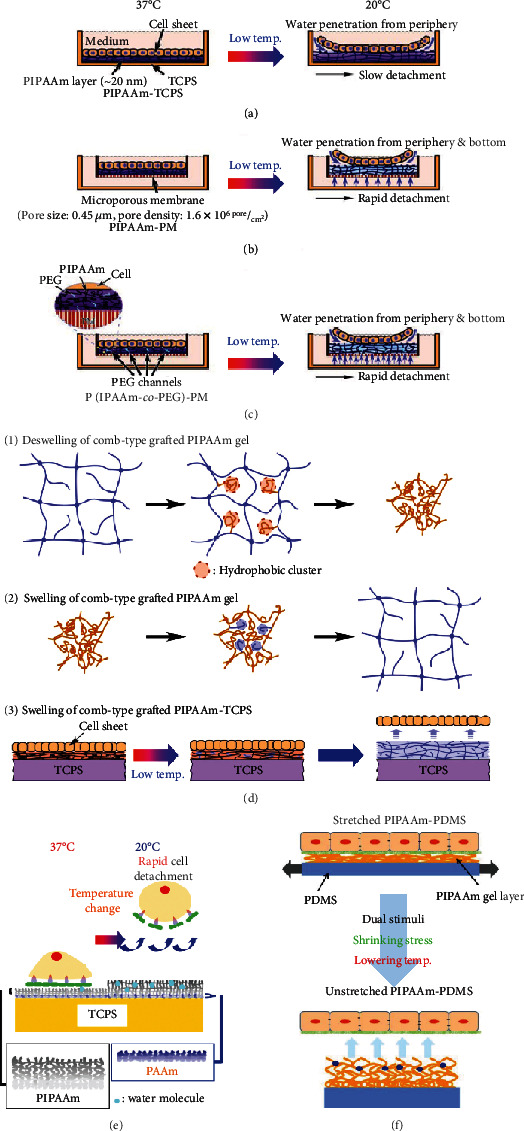
Schematic of temperature-responsive cell culture surface for acceleration of cell detachment. (a) PIPAAm-TCPS, (b) PIPAAm-PM, (c) P(IPAAm-*co*-PEG)-PM, (d) (1, 2) comb-type grafted PIPAAm gel and (3) ctPIPAAm-TCPS. These images of (a), (b), and (c) from reference [37] (Z. Tang et al., Temperature-Responsive Polymer Modified Surface for Cell Sheet Engineering, Polymers, 4, pp. 1478) are under a Creative Commons Attribution 3.0 International License (CC-BY-3.0). Illustration of (d) was reprinted with permission from reference [38]. Copyright © 2010, Elsevier Ltd. (e) PIPAAm-PAAm-TCPS with a double polymer layer and (f) stretchable temperature-responsive cell culture surface (PIPAAm-PAAm-TCPS). Illustration of (e) was reprinted with permission from reference [39]. Copyright © 2014, Acta Materialia Inc. Published by Elsevier Ltd.

Based on the concept that water molecules were efficiently supplied to the interface between the cell sheets and the PIPAAm-grafted surface to accelerate the hydration of PIPAAm chains below the cell sheet, PIPAAm gel was chemically deposited onto a porous membrane (PM) such as a cell culture insert (PIPAAm-PM) (Figures 3(a) and 3(b)) [32]. The original micropore structure was preserved in PIPAAm-PM after grafting the PIPAAm gel, enabling the ready supply of water molecules through the pores to the basal part of the cell sheet. Indeed, PIPAAm-PM showed quicker cell sheet detachment than PIPAAm-TCPS at temperatures lower than 20°C. In actual, PIPAAm-PM required ~30 min for detachment of cell sheet, while PIPAAm-TCPS did ~75 min (each cell culture area was 4.2 cm^2^).

Polyethylene glycol (PEG) chains and PIPAAm cografted PM (P(PIPAAm-*co*-PEG)-PM) further accelerated cell sheet detachment (Figure 3(c)) [33]. The incorporated PEG moiety allowed rapid water molecule diffusion from the periphery toward the center of the cell sheet as well as beneath one, accelerating cell sheet detachment in comparison with PIPAAm-TCPS and PIPAAm-PM.

The monomer newly synthesized by Aoyagi et al. [34], 2-carboxyisopropylacrylamide (CIPAAm), is structurally similar to *N*-isopropylacrylamide, besides the carboxyl group. IPAAm copolymerized with CIPAAm (P(IPAAm-*co*-CIPAAm)) showed almost the same LCST and temperature response character as PIPAAm in aqueous solution. The P(IPAAm-*co*-CIPAAm) gel-grafted TCPS (P(IPAAm-*co*-CIPAAm)-TCPS) exhibited cell adhesive characteristics similar to those of PIPAAm-TCPS and TCPS, whereas the cell sheet was more rapidly detached from P(IPAAm-*co*-CIPAAm) gel-grafted TCPS as the hydrophilic carboxyl group of CIPAAm accelerated the hydration of the grafted P(IPAAm-*co*-CIPAAm) gel [34].

### 3.2. Temperature-Responsive Cell Culture Surface Incorporated with Free Mobile PIPAAm Chains

The comb-type grafted PIPAAm gel, which contains free mobile PIPAAm chains, showed rapid deswelling by temperature change in comparison to a conventional PIPAAm cross-linked hydrogel (Figure 3(d), (1)) [35–37]. This is because the rapid dehydration of the free mobile PIPAAm chains forms hydrophobic clusters inside the comb-type PIPAAm hydrogel above the LCST. The hydrophobic cluster promotes dehydrating the PIPAAm chains in the gel, enabling quick shrinkage of the PIPAAm gel. Based on the concept that free mobile PIPAAm chains also enabled rapid swelling of PIPAAm hydrogels, comb-type PIPAAm gels were chemically deposited on the TCPS (ctPIPAAm-TCPS) using IPAAm and PIPAAm macromonomers (Figure 3(d), (2, 3)) [38]. At 37°C, cells adhere and spread on ctPIPAAm-TCPS as well as PIPAAm-TCPS. After confluency, the cell sheets were more rapidly detached from the ctPIPAAm-TCPS in comparison with PIPAAm-TCPS upon lowering temperature (20°C). As expected, the ctPIPAAm-TCPS containing free mobile PIPAAm chains attained more rapid cell sheet harvesting. The free mobile PIPAAm chains promoted hydration of the deposited comb-type PIPAAm gels.

### 3.3. PIPAAm Gel-Grafted Hydrophilic Polymeric Layer

A double polymeric gel layer consisting of nanoscale PIPAAm and polyacrylamide (PAAm) gels was grafted onto TCPS (PIPAAm-PAAm-TCPS) by successively grafting PAAm and PIPAAm (Figure 3(e)) [39]. The basal part of the double polymeric layer possibly formed an interpenetrating polymer network (IPN), which enabled the rapid swelling of the double polymeric gel layers, as the hydrophilic PAAm component not only provides water molecules but also facilitates hydration of PIPAAm chains close to the basal hydrophobic TCPS surface [40, 41]. The PIPAAm-PAAm-TCPS has double layers, where the hydrophilic PAAm gel layer is sandwiched between the basal TCPS and PIPAAm gel layer. Optimization of the amount of grafted PIPAAm and PAAm showed cell adhesion character similar to PIPAA-TCPS at 37°C and rapid cell sheet detachment by lowering temperature (20°C). However, cells were not attached to PIPAAm-PAAm-TCPS with graft PAAm more than 3.3 *μ*g/cm^2^, because the PAAm layer likely hydrates PIPAAm chains at its basal part, where IPN was formed.

### 3.4. Stretchable Temperature-Responsive Cell Culture Surface

For a conventional PIPAAm-TCPS as introduced above, the thickness of the grafted PIPAAm gel was dominated by the amount of applied IPAAm monomer. The thickness of grafted PIPAAm layer could not be modulated after the polymeric layer was grafted on hard base materials such as TCPS and glass. In contrast, as described above, cell attachment and detachment properties depend on the thickness of the graft polymeric layer. To readily modulate the thickness of the polymeric graft layer, as well as subsequent cell attachment and detachment events independent of the amount of applied monomer, PIPAAm gel grafted on an elastic polydimethylsiloxane (PDMS) surface (PIPAAm-PDMS) was prepared as a stretchable temperature-responsive cell culture surface *via* EB irradiation [42]. The grafted PIPAAm gel layer of PIPAAm-PDMS dynamically changed its thickness through the application of mechanical stress such as stretching and shrinking to the PIPAAm-PDMS. The PIPAAm layer was more hydrophobic due to thinning the graft polymeric layer when stretched, while the surface was more hydrophilic because of increasing the thickness of the layer when shrunken (Figure 3(f)). In reality, the uniaxially stretched PIPAAm-PDMS was more hydrophobic and more cell adhesive than unstretched PIPAAm-PDMS at 37°C. PIPAAm-PDMS showed a chemically stable surface due to the long-term temperature-dependent surface wettability change. Moreover, shrink of the stretched PIPAAm-PDMS followed by lowering the temperature (20°C) (dual stimulation) promoted cell detachment compared to only temperature change. Such a tendency was also observed in the cell sheet detachment process. Dual stimulation promoted cell sheet detachment from the stretched PIPAAm-PDMS surface.

## 4. Development of Various Methods for Fabrication of Temperature-Responsive Cell Culture Surfaces

Information regarding the thickness dependency of the grafted PIPAAm layer on cell adhesion behavior as described above has given valuable insights into the design and fabrication of temperature-responsive cell culture surfaces, possibly motivating some researchers to develop new fabrication methods [43]. Methods such as polymer (or polymeric gel particles) coating [44–53], photo-irradiation [54–57], and surface-initiated living radical polymerization methods such as atom transfer radical polymerization (ATRP) [58–63] and reversible addition-fragmentation chain transfer polymerization (RAFT) have been reported [64–66].

Surface-initiated ATRP and RAFT were attained to precisely control the length of PIPAAm (molecular weight) on the polystyrene and glass surface with dense PIPAAm chains. Intensive research on the PIPAAm brush surface demonstrated that the PIPAAm chain length and density of brushes substantially influence cell attachment and protein adsorption behaviors. When the molecular weight (Mn) and density of the graft polymeric chains were optimized to be 23,000–58,000 and 0.03–0.04 (chains/nm^2^), the PIPAAm brush surface enabled to express temperature-dependent cell attachment/detachment property. At the optimal conditions, more importantly, the temperature-dependent cell attachment/detachment property was due to the adsorbed fibronectin between the PIPAAm brushes, illustrated as ternary adsorption in previous reports [61, 63, 67].

Commercially available PIPAAm-TCPS (UpCell^®^) and conventional PIPAAm-TCPS were prepared *via* the EB irradiation method. However, most researchers cannot access special and expensive equipment as mentioned above for producing temperature-responsive cell culture surfaces. Some researchers also pointed out that the new method could potentially reduce the cost of commercially available PIPAAm-TCPS. Based on this, facile and cost-effective spin coating and UV or visible light irradiation methods have been developed for the production of temperature-responsive cell culture surfaces.

A block copolymer (poly(butyl methacrylate)-*b*-PIPAAm) (PBMA) (PBMA-*b*-PIPAAm) was physically coated on a polystyrene surface using a spin-coating method. Physically coated temperature-responsive polymers tended to be dissolved and eluted from the surface below the LCST [45, 52, 68], whereas, in the case of PBMA-*b*-PIPAAm-coated polystyrene surfaces, the hydrophobic PBMA units were strongly adsorbed on the surface through hydrophobic interactions, preventing coated PBMA-*b*-PIPAAm from elution from the surface below the LCST [44]. Optimizing the thickness of the coated PBMA-*b*-PIPAAm, PBMA-*b*-PIPAAm-coated surfaces enabled the expression of temperature-dependent cell attachment/detachment properties. PBMA-*b*-PIPAAm was also used for the preparation of a temperature-responsive cell culture insert [47].

The visible light irradiation method was also exploited for the easy fabrication of temperature-responsive cell culture surfaces [57]. The fabrication consists of two steps. Thioxanthone photo-initiator groups are immobilized on polystyrene (Th-PSt) dishes by incubating thiosalicylic acid dissolved in concentrated sulfuric acid. As a second step, an aqueous IPAAm solution containing *N*-methyldiethanolamine was added to the Th-PSt surface followed by irradiation of visible light. The resultant PIPAAm-grafted PSt surface exhibited temperature-dependent cell sheet recovery. These new methods would allow researchers to conveniently prepare temperature-responsive cell culture surfaces without using special and expensive equipment such as EB irradiation.

## 5. Temperature-Responsive Cell Culture Surfaces Equipped with Bioactive Ligands

**Figure 4:**
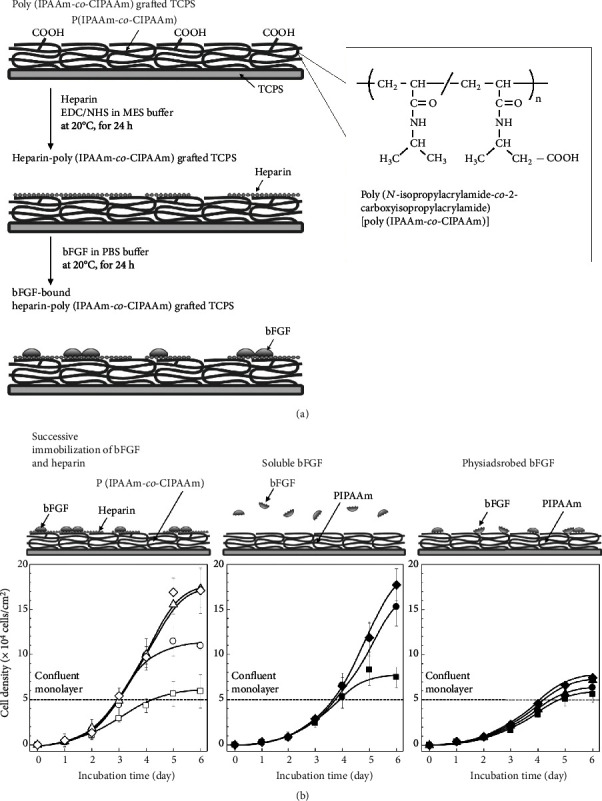
Schematic of (a) preparation of bFGF-Hep-P(IPAAm-*co*-CIPAAm)-TCPS and (b) time-courses of cell densities (NIH/3T3) on bFGF-Hep-P(IPAAm-*co*-CIPAAm)-TCPS (left), PIPAAm-TCPS with dissolved bFGF (middle), and bFGF physically adsorbed PIPAAm-TCPS (right). Reprinted with permission from reference [73]. Copyright © 2013, Elsevier Ltd.

Bioactive peptides and proteinaceous growth factors were chemically immobilized on P(IPAAm-*co*-CIPAAm)-TCPS *via* chemical reaction of the carboxyl groups of the surface with primary amine groups of the peptides [69, 70]. Ebara et al. immobilized cell adhesive molecules, RGDS peptides, on the P(IPAAm-*co*-CIPAAm)-TCPS, and demonstrated that human umbilical vein endothelial cells (HUVECs) were cultured at 37°C in the absence of serum [69]. After HUVECs reached confluency, they were recovered by reducing the temperature (20°C) [70]. These results demonstrated control of specific interactions between cultured cells and peptides. The P(IPAAm-*co*-CIPAAm) gel-grafted TCPS were also available for immobilizing heparin molecules, which have a specific affinity for various heparin-binding proteins (e.g., basic fibroblast growth factor (bFGF or FGF-2) (Figure 4(a)), heparin-binding epithelial cell growth factor (EGF)-like growth factor (HB-EGF), and VEGF [71–73]. Similar to heparan sulfate chains on proteoglycans, the immobilized heparin offers appropriate sites, which make growth factors form stable complexes. The growth factors can maintain their activities and prevent diffusion.

Successively immobilized heparin and bFGF molecules on P(IPAAm-*co*-CIPAAm)-TCPS (bFGF-Hep-P(PIPAAm-*co*-CIPAAm)-TCPS) showed faster proliferation of fibroblasts than physically adsorbed bFGF surface and dissolved bFGF in cell culture medium (Figure 4(b)) [73]. These results suggested that physically adsorbed bFGF molecules reduce the activity of the molecules due to their denaturation and/or unstable complex formation. Similarly, the heparin HB-EGF molecules were also successively immobilized on heparin-immobilized temperature-responsive cell culture surfaces [71, 72]. Arisaka and Kobayashi et al. demonstrated that HB-EGF-immobilized temperature-responsive cell culture surfaces were available for the fabrication of hepatocyte sheets. The hepatocyte sheet expressed genes specific for hepatocytes (albumin (*Alb*), hepatocyte nuclear factor 4 alpha (*Hnf4a*), coagulation factor IX (*F9*), and coagulation factor VII (*F7*)) during culture [72]. The specific gene expression was higher than in hepatocyte sheets cultured in the presence of dissolved HB-EGF. The hepatocyte sheet was detached from the surface at 20°C. Hepatocyte sheets could be useful as new therapeutic approach for treatment of liver disease, hemophilia [74].

## 6. Cell Sheet Manipulation Technology

Cell sheets generally shrink, wrinkle, and fold during detaching themselves from PIPAAm-TCPS. Such deformed cell sheets are troublesome for the manipulation and fabrication of cell-dense and thick tissue through layering and transplantation to native tissues and organs. To attain ideal manipulation and transplantation without deformation, commercially available products such as chitin membrane, porous PET, and hydrophilic modified poly(vinylidene difluoride) membranes were used as cell sheet carrier materials [75, 76]. These membranes were physically overlaid on cultured cells at 37°C after aspirating almost the entire culture medium (retaining a small amount). By reducing the temperature below the LCST, the cell sheet was detached and attached to the overlaid cell sheet carrier membrane. By gently peeling off the membrane from the PIPAAm-TCPS with tweezers, the detached cell sheet could be lifted up and manipulated with the membrane without shrinking and wrinkling. After transferring the cell sheet onto another cell sheet or biological tissue or organ, the cell sheet was released from the membrane with the addition of some medium. Additionally, commercially available membranes, synthetic polyion complex, poly(*N,N*-dimethylacrylamide-*co*-2-acrylamido-2-methylpropane sulfonic acid) (P(DMAAm-*co*-AMPS)) and poly(*N,N*-dimethylacrylamide-*co*-2-acryloxyethyltrimethylammonium chloride) (P(DMAAm-*co*-AETA-Cl)), were also exploitable as cell sheet carriers [77].

**Figure 5:**
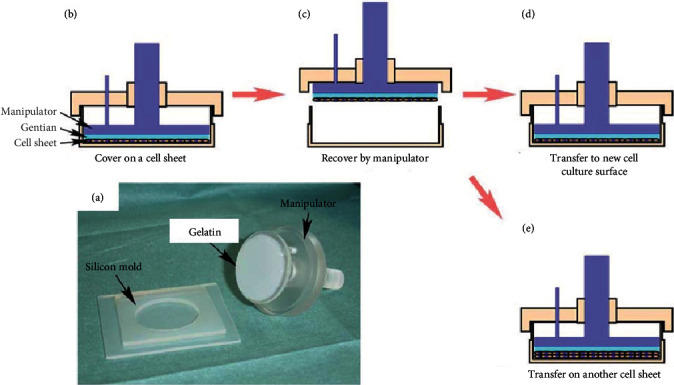
Schematic of recovery, harvesting, transferring, and layering cell sheet using the gelatin-coated plunger device. (a) Macroscopic view of gelatin-coated plunger-like device as a cell sheet manipulator. (b) Cell sheet was covered by the gelatin-coated device and incubated at 20°C. (c) Cell sheet was harvested by the device. (d) The harvested cell sheet was transferred to a new cell culture surface. (e) The harvested cell sheet was layered on another cell sheet. The images from reference [36] (Z. Tang and T. Okano., Recent development of temperature-responsive surfaces and their application for cell sheet engineering, Regen Biomater, 2014, 1, pp. 91-102) are under a Creative Commons Attribution 4.0 International License (CC-BY-4.0).

In addition to the artificial polymeric membrane, gelatin gel was also used as a cell sheet carrier. Gelatin was coated on a plunger-like device that aimed at being a cell sheet manipulator (Figure 5) [78, 79]. When the gelatin-coated device was deposited on confluent cells cultured on PIPAAm-TCPS at 20°C (Figure 5(b)), the cell sheet was detached from the surface and preferentially adhered to the gelatin-coated device. It was lifted from the device and placed on another cell sheet cultured on PIPAAm-TCPS (Figure 5(c)). Afterward, reducing the temperature below LCST produced a double-layered cell sheet structure (Figure 5(d)). Repeating the process of layering the cell sheet with the device enabled the fabrication of dense and thick 3D tissue (Figure 5(e)). Additionally, a combination of the manipulation technique for cell sheet and robot technology successfully developed the automatic cell sheet stacking system, which enabled the automatic fabrication of five-layered human skeletal muscle myoblast sheets (70–80 *μ*m thickness) in 100 min [80]. This automation technology contributed to the development of a flexible and automated manufacturing facility: a tissue factory, which consists of nine moduli and a material preparation isolator [81]. The tissue factory automatically manufactures a five-layered human myoblast sheet as a cell-based health care product.

## 7. Cell Sheet-Based Regenerative Medicine

**Figure 6:**
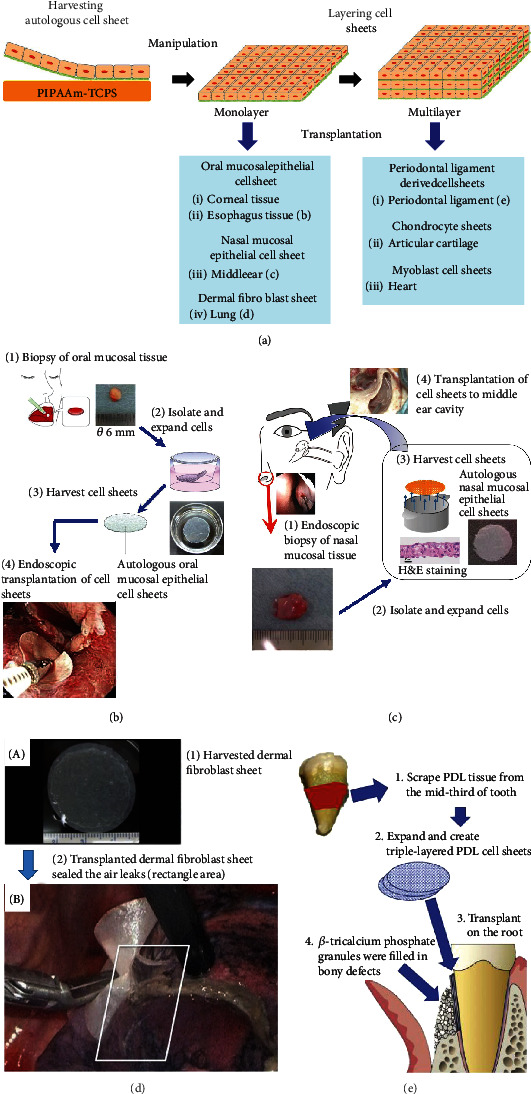
Clinical applications of autologous cell sheet-based regenerative medicine. (a) Schematic of harvesting, manipulating, and transplanting mono- and multilayered autologous cell sheets for regeneration of various types of human tissues and organs. (b) Transplantation of autologous oral mucosal epithelial cell sheet for treatments of damaged artificial ulceration after esophageal ESD. This illustration of (b) was adopted from reference [13] with permission form AGA Institute. Published by Elsevier Inc. (c) Transplantation of autologous nasal mucosal epithelial cell sheet for regeneration of postoperative mucosal regeneration of the middle ear. This image from reference [17] (K. Yamamoto et al., Middle ear mucosal regeneration by tissue-engineered cell sheet transplantation, 2017, npj Regenerative Medicine, 2, pp. 6) is licensed under a Creative Commons Attribution 4.0 International License (CC-BY-4.0). (d) Transplantation of autologous dermal fibroblast sheet for sealing the air leaks [19]. This image from reference [19] (M. Kanzaki et al., Bio-artificial pleura using an autologous dermal fibroblast sheet, 2017, npj Regenerative Medicine, 2, pp. 26) is licensed under a Creative Commons Attribution 4.0 International License (CC-BY-4.0). (e) Transplantation of three-layered autologous PDL tissue derived cell sheets on the root of the damaged tooth. This image was adopted from reference [15] with permission from The Japanese Society for Regenerative Medicine. Production and hosting by Elsevier B.V.

Cell sheet engineering has drawn attention, becoming a promising treatment for damaged tissues and organs. Cell sheet transplantation was superior to cell injection approaches in terms of the resultant survival rate of cells and the subsequent recovery of damaged tissues and organs [82]. Transplanted cell sheets can adhere to biological tissues *via* their deposited ECM and survive thereon. However, injected cells did not stay on the tissues because they were scattered. Clinical application of autologous cell sheets has been performed, demonstrating successful treatment of various damaged human tissues and organs (Figure 6(a)).

Nishida et al. pioneered corneal epithelial regeneration treatment using a monolayer cell sheet. From tissues including epithelial stem cells derived from patients who lost eye vision due to alkali burn or drug side effects, corneal epithelial cells were expanded and recovered as a monolayer cell sheet from PIPAAm-TCPS [12, 83]. The corneal epithelial cell sheet was transplanted to treat the injured eye. For binocular diseases, oral mucosal cell sheets were alternatively available for treatment of patient eyes [12, 83]. Their ability to see was improved after the cell sheet transplantation. Researchers in France also reported the efficacy of oral mucosal cell sheet transplantation for regeneration of the ocular surface in patients with binocular eye diseases [84].

Endoscopic submucosal dissection (ESD) is conducted as a less-invasive approach for resection of esophageal cancer than a surgical one. However, after ESD, esophageal stenosis frequently occurs due to artificial ulcer scarring, lowering the quality of life. Ohki et al. developed an approach for the regeneration of cell sheet-based esophagus tissue after EDS treatment [13]. They endoscopically transplanted oral mucosal epithelial cell sheets onto the ulcer surface (Figure 6(b)). The transplanted cell sheet promotes reepithelialization of the esophagus and suppresses the stenosis event [14]. The cell sheet transplantation is available for treatment of patients with Barrett's esophagus [85].

After surgical treatment of adhesive otitis media and cholesteatoma middle ear, early postoperative mucosal regeneration of the middle ear and the mastoid cavity is desired for structural and functional reconstruction of the cavity as mucosal regeneration prevents readhesion of the tympanic membrane and recurrence of adhesive media and cholesteatoma. Yamamoto et al. have isolated nasal mucosa epithelial cells from the mucosal tissue of patients undergoing endoscopy and fabricated autologous nasal mucosa epithelial cell sheets using temperature-responsive cell culture inserts (Figure 6(c)) [17]. By combing tympanoplasty and autologous nasal mucosal epithelial cell sheet transplantation, they developed a new treatment method for postoperative mucosal regeneration of the middle ear. The epithelial cell sheets were transplanted to the damaged middle ear cavity, where mucosa was lost. Transplantation successfully prevents the reappearance of cholesteatoma, tympanic membrane adhesion, and tympanic membrane retraction.

Air leaks after pulmonary resections can produce severe complications without proper treatment. Kanzaki et al. applied autologous dermal fibroblasts cells, which were isolated from patient skin tissue, followed by an expansion on temperature-responsive cell culture surface, to close a patient's pleural defect. The air leaks were completely sealed with the cell sheet (Figure 6(d)) [19].

Cells from periodontal ligaments on wisdom teeth were cultured and harvested as periodontal ligament cell sheets. Once periodontal tissues are damaged, it is difficult to heal spontaneously. Periodontal disease causes the loss of periodontal tissue, causing the instability or loss of teeth. After expanding and culturing cells isolated from periodontal ligament (PDL) tissue of patients, three-layered autologous PDL-derived cell sheets were transplanted to root surface of patients with bone prosthetic materials (Figure 6(e)) by Iwata and Ishikawa et al. [15, 16]. As a result, transplantation not only stabilizes teeth but also recovers periodontal ligament tissue, reduces the periodontal probing depth, and improves the bone around the transplanted cell sheets.

Damaged articular cartilage cannot heal spontaneously due to a lack of blood supply and a low density of cells. Sato et al. developed a new treatment for damaged articular cartilage [18]. Chondrocytes and synovial cells collected from patients were cocultured on temperature-responsive cell culture inserts and cell culture surfaces, respectively. Layered chondrocyte sheets were transplanted to cartilage defects of patients that were treated with conventional surgical treatment. This demonstrates that the transplantation promotes hyaline cartilage repair.

Sawa et al. have transplanted autologous skeletal myoblast sheet for the regeneration of ischemic myocardium [11]. Skeletal myoblast cells were isolated from muscle tissues of patient leg and cultured for the fabrication of autologous skeletal sheets. In a first clinical trial, they transplanted three-layered autologous skeletal myoblast sheets onto the heart surfaces of a patient with serious cardiac insufficiency due to dilated cardiomyopathy. Cell sheet transplantation improved cardiac function in the patient. Later, it also enabled the removal of a left ventricular assist device from the patient. The improvement of cardiac function after cell sheet transplantation is attributable to cytokine secretion (such as VEGF, FGF, and HGF) from the transplanted cell sheets and paracrine effects inducing the recruitment of stem cells. Sawa et al. treated more patients with myoblast cell sheet transplantation. They demonstrated the feasibility and safety of transplantation of autologous skeletal myoblast sheets in patients with severe chronic disease [86]. Based on these insights, “Heart Sheet” was released as a medical product for patients with ischemic heart disease from Terumo Corporation, Japan in 2016.

## 8. Conclusions

Temperature-responsive cell culture surface established cell sheet engineering, which has offered fundamental technology for the fabrication of 3D tissues and organs as well as cell sheet-based regenerative medicine. Precise control of the nanoscale graft PIPAAm layer is essential for expression of temperature-dependent cell attachment/detachment character and subsequent cell sheet recovery. Considerable progress in cell sheet engineering for years has created new and various types of temperature-responsive cell culture surfaces, automated fabrication and manipulation systems, and new medical treatments for damaged human tissues and organs. Currently, iPS and mesenchymal stem cells (MSCs) have become attractive cell sources for cell-based therapy and have been intensively researched in basic and clinical research [87–90]. Transplantation of allogenic cell sheets using MSCs has been conducted as a basic study for the development of new medical treatments. Various types of cells differentiated from iPSCs and MSCs would extend the range of application of cell sheet-based therapies for damaged tissues and organs: salivary glands [91], diabetic nephropathy (chronic kidney disease) [92, 93], cerebral ischemia [94], and so on, [95–97] with more complicated structures and functions.
